# Anti-SARS-CoV-2 antibody levels and kinetics of vaccine response: potential role for unresolved inflammation following recovery from SARS-CoV-2 infection

**DOI:** 10.1038/s41598-021-04344-y

**Published:** 2022-01-10

**Authors:** F. Gianfagna, G. Veronesi, A. Baj, D. Dalla Gasperina, S. Siclari, F. Drago Ferrante, F. Maggi, L. Iacoviello, M. M. Ferrario

**Affiliations:** 1 Research Center in Epidemiology and Preventive Medicine (EPIMED), Department of Medicine and Surgery, University of Insubria, Varese, Italy; 2 Mediterranea Cardiocentro, Napoli, Italy; 3 Department of Medicine and Surgery, University of Insubria, Varese, Italy; 4 Laboratory of Microbiology, ASST Sette Laghi, Varese, Italy; 5 Medical Center, ASST Sette Laghi, Varese, Italy; 6 Unit of Occupational and Preventive Medicine and Toxicology, ASST Sette Laghi, Varese, Italy; 7 Department of Epidemiology and Prevention, IRCCS Neuromed, Pozzilli, Italy

**Keywords:** Predictive markers, Viral infection, Epidemiology, Risk factors, RNA vaccines

## Abstract

The immune response after SARS-CoV-2 vaccine administration appears to be characterized by high inter-individual variation, even in SARS-CoV-2 positive subjects, who could have experienced different post-infection, unresolved conditions. We monitored anti-SARS-CoV-2 IgG levels and kinetics along with circulating biomarkers in a cohort of 175 healthcare workers during early immunization with COVID-19 mRNA-LNP BNT162b2 vaccine, to identify the associated factors. Subjects with a previous SARS-CoV-2 infection were characterized by higher BMI and CRP levels and lower neutrophil count with respect to naïve subjects. Baseline IgG levels resulted associated with CRP independently on BMI and inflammatory diseases. Among 137 subjects undergoing vaccination and monitored after the first and the second dose, three kinetic patterns were identified. The pattern showing a rapid growth was characterized by higher IgG levels at baseline and higher CRP and MCHC levels than negative subjects. Subjects previously exposed to SARS-CoV-2 showed higher levels of CRP, suggesting persistence of unresolved inflammation. These levels are the main determinant of IgG levels at baseline and characterized subjects belonging to the best performing, post-vaccine antibody kinetic pattern.

## Introduction

The ongoing COVID-19 pandemic caused by SARS-CoV-2 has resulted in hundreds of millions of infections and millions of deaths worldwide^1^, along with social and economic problems. New SARS-CoV-2 variants are associated with high transmission and rising numbers of COVID-19 cases are overwhelming global health care capacity^2^. Beyond social distancing, widespread use of face masks, hand hygiene, and timely isolation of infected subjects that helped to reduce viral circulation within the community^3^, since the end of 2020 vaccines anti-SARS-CoV-2 became available and markedly contributed to the decline in the infection incidence^4^. Despite the considerable efficacy of the available vaccines in reducing infection, there are some unresolved issues.

Since the number of vaccine doses was inadequate to meet current global needs, revisiting vaccination strategies became a priority^5^. A lower dosage was suggested for subjects who have recovered from the natural infection since they are thought to have protective immunity, with long-lasting B and T memory responses^6^ despite a short-lasting plasma cells production of neutralizing antibodies^7^. Moreover, the first studies on vaccine response confirmed a robust T-cell response after a single dose of vaccine^8^ in previously exposed to SARS-CoV-2, along with a more pronounced antibody response with respect to unexposed^9^. However, ideal vaccine dosing regimens, able to optimize the resource and minimize the risk of delaying a protective immunity, as well as the target population who could benefit from dose reduction, are not yet known.

Similar to the clinical response to the SARS-CoV-2, the immune response after vaccine administration appears to be characterized by high inter-individual variation^10^. The identification of markers of immunogenicity can help to improve vaccine development and personalized vaccination strategies^11–13^. Host-specific factors associated with the efficacy of anti-SARS-CoV-2 vaccines are not yet clearly outlined, although they may include all factors affecting immune system functionality, such as, beyond genetics, age, sex, psychosocial factors, lifestyles, microbiota, drugs, clinical history, and other previous vaccinations, as well as a basal inflammatory state^14^, as confirmed by recent studies^15–18^. As expected, subjects with previous exposure to SARS-CoV-2 show the highest response. However, immunity among patients recovered from SARS-CoV-2 infection is still poorly described^19^ and it is not yet clear if the level of the elicited vaccine response as well as the pre-vaccine antibody titer are determined by potential changes due to previous SARS-CoV-2 infection, as an unresolved basal inflammatory state^20^.

We established a longitudinal cohort of 175 healthcare workers in an Italian healthcare company to assess circulating anti-SARS-CoV-2 S1/S2 specific antibodies at baseline and throughout first and second immunization with the BNT162b2 mRNA COVID-19 vaccine (Comirnaty®; Pfizer-BioNTech), as well as to identify their potential determinants. Specifically, we aimed to investigate the prevalence of the previous infection, the IgG levels before (the day before the date of vaccination—T0) and after vaccination (the day before the 2nd dose—T1, and 2 weeks after the 2nd dose—T2), then the early kinetic patterns of serological responses. Finally, we investigated the characteristics of subjects associated with prior exposure to the virus, with the IgG levels and with the best performing kinetic pattern.

## Results

### Baseline characteristics

Among 175 participants, a total of 48 (27.4%) subjects had a previous positivity to RT-PCR SARS-CoV-2 and 9 of them (18.8%) showed negative IgG antibody levels. Conversely, among subjects without a known previous SARS-CoV-2 infection, 27 (21.3%) subjects showed positive antibody levels. The total of subjects being positive either in a previous RT-PCR test or to antibodies was 75 (42.9%) subjects. Baseline characteristics of the 175 participants according to SARS-CoV-2 positivity are provided in Table 1.

**Table 1:** Population characteristics by previous SARS-CoV-2 positivity and IgG levels at baseline (N = 175).

	Negative RT-PCR		Positive RT-PCR		*p* value^b^	*p* value^c^
	A. IgG negative	B. IgG positive	C. IgG positive	D. IgG negative		
N	100	27	39	9	–	–
Baseline anti-S1/S2 IgG (AU/ml)*	4.5 (3.8, 7.5)	27 (17, 66)	50 (28, 104)	3.8 (3.8, 4.9)	< .0001	< .0001
**Demographic and clinical history**						
Age (years)	47.5 (8.3)	50 (7.8)	47 (9.6)	47.7 (11.9)	0.65	0.64
Women	90 (90%)	25 (92.6%)	34 (87.2%)	6 (66.7%)	0.18	0.49
Body mass index (kg/m^2^)*	22.9 (20.3, 27)	23.4 (20.9, 25.6)	24.7 (22.5, 27.5)	25.9 (23.9, 26.5)	0.01	0.03
Anti-tetanus vacc. (last 10 years)	19 (19%)	3 (11.1%)	5 (12.8%)	2 (22.2%)	0.63	0.32
Anti-influenza vacc. (last year)	31 (31%)	12 (44.4%)	5 (12.8%)	3 (33.3%)	0.02	0.53
BCG vaccination or PPD positivity	38 (38%)	12 (44.4%)	10 (25.6%)	2 (22.2%)	0.06	0.41
Autoimmune diseases	12 (12%)	2 (7.4%)	8 (20.5%)	2 (22.2%)	0.10	0.45
Chronic diseases^a^	19 (19%)	2 (7.4%)	6 (15.4%)	0 (0%)	0.48	0.13
**Organ damage biomarkers**						
Creatinine (mg/dl)	0.9 (0.1)	0.8 (0.1)	0.9 (0.1)	0.9 (0.1)	0.84	0.36
Aspartate transam.—AST (U/l)	21.6 (6.8)	24.2 (8.2)	24.2 (6.8)	21 (7.1)	0.25	0.05
Alanine transam.—ALT (U/l)	24.6 (15)	27.6 (16)	27.3 (13.6)	26.9 (12.4)	0.43	0.22
Lactate dehydrogenase—LDH (U/l)	190 (33)	201.2 (29.8)	200.8 (42.5)	190.6 (20.6)	0.29	0.08
Troponin T (ng/l)	4.3 (4.1)	4.7 (2.9)	4.2 (1.9)	4.6 (2.1)	0.77	0.85
**Inflammatory biomarkers**						
CRP (mg/l)	1.9 (1.9)	2.5 (2.8)	3.9 (5.5)	2.2 (1.6)	0.01	0.01
Leucocytes (10^9^ cells/l)	7.1 (1.7)	6.8 (2.3)	6.2 (1.6)	6.4 (1.3)	0.01	0.02
Lymphocytes (10^9^ cells/l)	2.2 (0.7)	2.1 (0.8)	2 (0.6)	1.9 (0.5)	0.12	0.16
Monocytes (10^9^ cells/l)	0.5 (0.1)	0.5 (0.2)	0.5 (0.2)	0.5 (0.1)	0.55	0.81
Neutrophils (10^9^ cells/l)	4.2 (1.3)	4 (1.7)	3.5 (1.3)	3.9 (1.2)	0.01	0.02
Platelets (10^9^ cells/l)	266.4 (55.1)	273.7 (51.3)	264.6 (60.5)	263 (54.8)	0.70	0.88
Mean platelet volume—MPV (fl)	10.7 (0.8)	10.7 (0.9)	10.6 (0.9)	10.4 (0.7)	0.41	0.41
**Red blood cells**						
Red blood cells (10^12^ cells/l)	4.7 (0.5)	4.7 (0.4)	4.7 (0.5)	4.9 (0.4)	0.88	0.88
Mean corp. volume—MCV (fl)	87.6 (5.5)	88.1 (5.1)	85.9 (6.9)	89.2 (2.9)	0.23	0.56
Hematocrit (%)	41 (3.4)	41.1 (3.9)	39.9 (4.6)	43.6 (3)	0.56	0.71
Red distribution width—RDW (%)	13 (1)	13.2 (1)	13.5 (2)	12.5 (0.8)	0.22	0.19
Hemoglobin (g/dl)	13.8 (1.3)	13.7 (1.4)	13.4 (1.9)	14.8 (1.3)	0.64	0.51
Mean corp. hem. conc.-MCHC (g/dl)	33.7 (1)	33.3 (0.9)	33.5 (1.6)	33.9 (0.8)	0.75	0.15

Mean (SD) for continuous variables, n (%) for categorical variables.

Abbreviations: *PPD* purified protein derivative test (tuberculin test).

^a^Diabetes mellitus, chronic renal failure, hypothyroidism.

In the Table: either mean (SD) or median (25–75°percentiles, as indicated with an *) for continuous variables; and n (%) for categorical variables. *p* value: comparison across study populations, as defined below. Continuous variables: t-test (in occurrence of mean and SD) or Wilcoxon rank test (median and IQR). Categorical variables: chi-square tests.

^b^Comparison between negative (n = 127) and positive (n = 48) real-time RT-PCR [columns A and B vs. C and D].

^c^Comparison between negative real-time RT-PCR with negative Ab (n = 100), and either positive Ab or positive real-time RT-PCR (n = 75) [column A vs. B, C and D].

With respect to negative, subjects positive to any of the two tests showed higher body mass index (BMI) and CRP levels and lower neutrophil levels (all *p* < 0.01; Table 1). Among positive subjects, continuous levels of anti-SARS-CoV-2 IgG at baseline were associated with CRP (0.33 ± 0.12, *p* = 0.007) and red blood cell parameters (Supplementary Table 1). For CRP (Supplementary Fig. 1), results remain unchanged using BMI as a covariate and excluding subjects with autoimmune diseases. Moreover, the estimates remain unchanged when restricting the analysis to those with or without a history of symptoms, although statistical significance disappeared due to limited sample size (n = 29). No associations were found between positivity and occupational characteristics such as professional categories or having worked in a unit with COVID-19 patients. Considering positivity to a previous RT-PCR test only, positive subjects were also less probably vaccinated for influenza during the last year (*p* = 0.02) and, although non-significantly, showed a lower prevalence of previous Bacille Calmette-Guérin (BCG) vaccination or tuberculosis (TB) infection (*p* = 0.06; Table 1).

**Figure 1:**
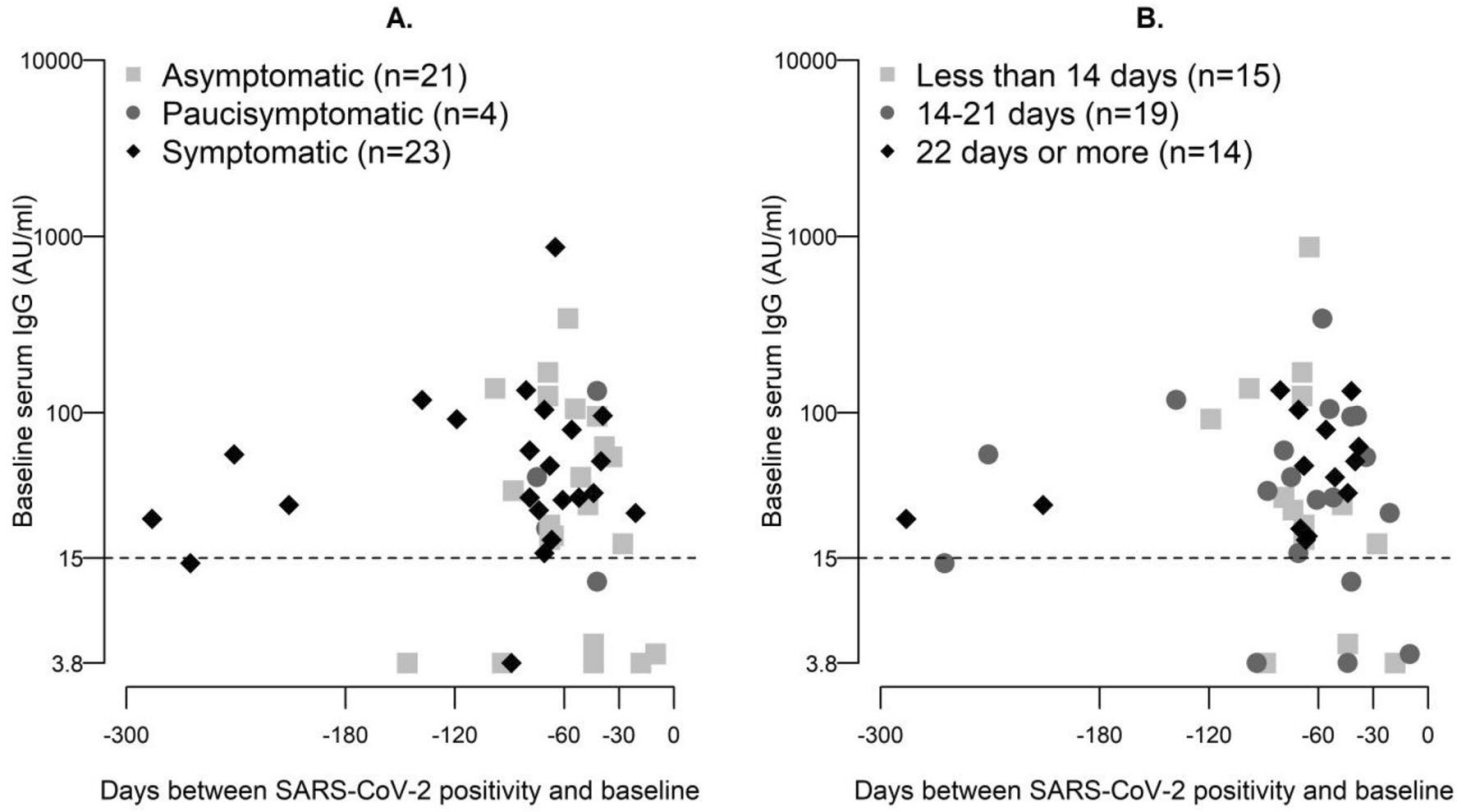
Baseline IgG levels and time distance (days) between measurement and SARS-CoV-2 first negative nasal swab among subjects previously positive to RT-PCR, stratified by (panel **A**) history of symptoms and (panel **B**) positivity duration (N = 48).

Among subjects with a previous SARS-CoV-2 positivity, no differences in baseline characteristics were found between those with and without measurable antibody levels (data not shown). Considering the characteristics related to the SARS-CoV-2 positivity, we found no differences neither in days between RT-PCR positivity and baseline (median days − 68 vs. − 44, for subjects with and without measurable antibody levels, respectively; Wilcoxon rank test p value = 0.76) nor in positivity duration. However, none of the subjects with positivity to real-time RT-PCR more prolonged than 3 weeks (n = 14) resulted in IgG-negative (Fig. 1).

### Antibody kinetics following vaccination

**Figure 2:**
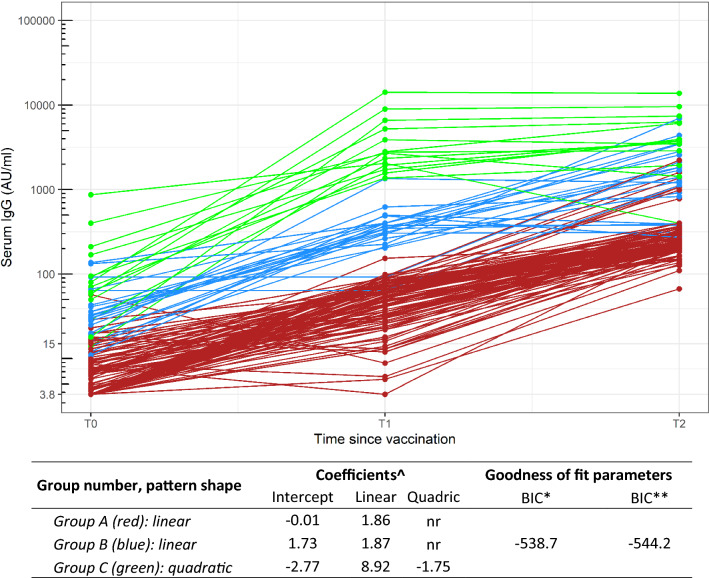
Antibody levels following vaccination (baseline—T0, 20 days after first dose—T1, 14 days after 2nd dose—T2): pattern shape, coefficients and goodness-of-fit parameters for the sub-population groups identified from trajectory analyses (best model), in the overall sample with repeated serum IgG measurements at T0, T1 and T2 (n = 137). Y log scale. ^: On the log-transformed serum IgG variable; *nr* not relevant (linear model); *: Bayesian Information Criterion, on the number of observations (n = 137 unique subjects, 3 time measurement each); **: Bayesian Information Criterion, on the number of unique subjects (n = 137 unique subjects).

A total of 137 subjects underwent vaccination within the study period, showing characteristics similar to the whole sample (Supplementary Table S2). Baseline IgG-positive subjects were 43 (mean ± SE 78.0 ± 141.5 AU/ml). Although 85% of subjects resulted positive at T1 (n = 127, 563.3 ± 1729.5 AU/ml), all subjects showed measurable IgG with a good serological response at T2 (136, 1041.3 ± 1933.8 AU/ml) except a subject in treatment with ocrelizumab^18^. Figure 2 shows early trajectories of antibody levels following vaccination. The pattern analysis identified three principal groups of subjects who shared a similar trajectory following vaccination: a first group (Group A, n = 101 subjects) with negative baseline IgG levels and a linear growth velocity; a second group (Group B, n = 22) with low baseline IgG levels (median 31 AU/ml, IQR:19–43 AU/ml) and a linear growth; a third group (Group C, n = 14) with higher antibody levels at baseline (median 75 AU/ml, IQR 50–169 AU/ml) and rapid growth following a quadratic function. The IgG levels at T1, rather than T2, resulted the main discriminant characteristic, showing no overlap between their distributions (means and 95%CI: Group A, 50.6 AU/ml, 45.5–55.7 AU/ml; Group B, 378.1 AU/ml, 266.6–489.6 AU/ml; Group C, 4158 AU/ml, 2068–6248 AU/ml).

Characteristics of subjects belonging to the 3 groups are shown in Table 2 and Supplementary Table 3. Several variables resulted statistically different among groups, mainly among markers of inflammatory status (CRP, WBC) or red blood cell biomarkers (mean corpuscular haemoglobin concentration—MCHC, distribution width of red blood cell volume—RDW). Some of them were also associated with previous SARS-CoV-2 positivity. We verified whether there were differences between Groups B and C, both constituted by subjects with a previous positivity to SARS-CoV-2 (34 out of 36, Supplementary Table 3) but with different baseline IgG levels and different growth velocity. No statistically significant differences were found. However, in a multivariate model including only variables associated with *p* < 0.01, there was an increasing trend of probability to belong to Group B (low baseline level) and C (high baseline levels and rapid growth) as compared to Group A (negative baseline levels) for CRP (for 1 SD: Group B, OR = 3.4, 95%CI: 1.8–6.4; Group C, OR = 4.6, 95%CI:2.2–9.9). Group C was also associated with older age (OR = 7.5, 95%CI:1.9–29.2) and lower levels of MCHC (OR = 0.29, 95%CI:0.13–0.61). The results remain unchanged, excluding subjects with autoimmune diseases, naïve subjects, and positive subjects without a history of symptoms.

**Table 2:** Characteristics of subjects undergoing SARS-Cov-2 vaccination according to kinetic pattern (n = 137).

Label	Group A (No IgG, linear increase)	Group B (low IgG, linear increase)	Group C (High IgG, quadratic increase)	*p* value
N	101	22	14	–
Baseline anti-S1/S2 IgG (AU/ml)*	5 (3.8, 8.9)	31 (19, 43)	75.5 (50, 169)	< .0001
**Demographic and clinical history**				
Age (years)	47.5 (8.8)	43.6 (9.4)	52.9 (8.6)	0.01
Women	89 (88.1%)	19 (86.4%)	13 (92.9%)	0.8
Previous SARS-CoV-2 Pos.—RT-PCR (%)	10 (9.9%)	16 (72.7%)	10 (71.4%)	< .0001
Body mass index (kg/m^2^)*	23.2 (20.3, 27.3)	24.2 (21.5, 26.5)	24.5 (23.7, 28.3)	0.24
Anti-tetanus vaccination (last 10 years)	18 (17.8%)	1 (4.5%)	1 (7.1%)	0.20
Anti-influenza vaccination (last year)	35 (34.7%)	5 (22.7%)	6 (42.9%)	0.42
BCG vaccination or PPD positivity	36 (35.6%)	7 (31.8%)	4 (28.6%)	0.84
Autoimmune diseases	12 (11.9%)	2 (9.1%)	4 (28.6%)	0.18
Chronic diseases^a^	16 (15.8%)	3 (13.6%)	4 (28.6%)	0.45
**Organ damage biomarkers**				
Creatinine (mg/dl)	0.9 (0.1)	0.9 (0.1)	0.8 (0.1)	0.64
Aspartate transaminase—AST (U/l)	22 (7.4)	22.6 (6.8)	24.3 (7.3)	0.55
Alanine transaminase—ALT (U/l)	25.4 (15.3)	27.2 (15.3)	28.6 (18.6)	0.72
Lactate dehydrogenase—LDH (U/l)	191 (33.1)	212 (46.5)	188.7 (32.8)	0.06
Troponin T (ng/l)	4.4 (4.1)	3.9 (1.2)	6 (4)	0.27
**Inflammatory biomarkers**				
CRP (mg/l)	1.9 (1.8)	4.8 (4.1)	5.3 (7.8)	< .0001
Leucocytes (10^9^ cells/l)	7.1 (1.8)	6.3 (1.3)	5.9 (2.1)	0.02
Lymphocytes (10^9^ cells/l)	2.2 (0.7)	1.8 (0.6)	1.8 (0.8)	0.05
Monocytes (10^9^ cells/l)	0.5 (0.2)	0.6 (0.2)	0.5 (0.2)	0.15
Neutrophils (10^9^ cells/l)	4.2 (1.4)	3.7 (1.1)	3.4 (1.6)	0.05
Platelets (10^9^ cells/l)	268.1 (53.1)	264.6 (65.3)	252.3 (56)	0.62
Mean platelet volume—MPV (fl)	10.7 (0.8)	10.7 (1.1)	10.6 (0.6)	0.94
**Red blood cells**				
Red blood cells (10^12^ cells/l)	4.7 (0.5)	4.8 (0.5)	4.6 (0.6)	0.75
Mean corpuscular volume—MCV (fl)	87.4 (5.4)	85.9 (7)	86.1 (8.6)	0.47
Hematocrit (%)	41.1 (3.5)	41 (5.2)	39.8 (5.3)	0.55
RDW (%)	12.9 (0.9)	13.5 (2)	14.1 (2.2)	0.003
Hemoglobin (g/dl)	13.9 (1.3)	13.8 (2.1)	13.1 (2.2)	0.22
MCHC (g/dl)	33.8 (1)	33.5 (1.3)	32.7 (1.7)	0.006

Abbreviations: *PPD* purified protein derivative test (tuberculin test).

^a^Diabetes mellitus, chronic renal failure, hypothyroidism.

In the Table: either mean (SD) or median ((25–75°percentiles, as indicated with an *) fpr continuous variables; and n (%) for categorical variables.

*p* value: comparison across study populations, as defined below. Continuous variables: t-test (in occurrence of mean and SD) or Wilcoxon rank test (median and IQR). Categorical variables: chi-square tests.

## Discussion

In our cohort of 175 healthcare workers, we found 42.9% of subjects previously infected with SARS-CoV-2, who were those with higher BMI and CRP levels and lower neutrophil count. IgG levels at baseline resulted associated with several red blood cell parameters, as well as with CRP independently on BMI and inflammatory diseases. In the subgroups of subjects undergoing SARS-CoV-2 mRNA vaccination, we identified three main antibody kinetic patterns, characterized by different baseline IgG levels (negative, low, high); the first two groups shared the same growth velocity, while the third one showed faster growth. High CRP and low MCHC levels characterized subjects within the third group with high baseline IgG levels and rapid vaccine response.

The SARS-CoV-2 seroprevalence found in our population of healthcare workers, as well as the percentage of the unknown history of SARS-CoV-2 infection, were in line with the literature^21^, taking into account the differences in recruitment settings (time, geographic region and levels of exposure of the recruited healthcare workers) among the published studies. A recent study on a Mediterranean population^22^, evaluating SARS-CoV-2-IgG antibodies in a large sample of hospital personnel found a seroprevalence of 11.0%, with important variation in percentage depending on the regional COVID-19 incidence and on professional categories considered, at different level of exposure risk. Similarly, we found 9 subjects with a previous exposure to SARS-CoV-2 who became negative for IgG and they were not those with a longer lag time by positivity diagnosis, as expected. Several studies showed a decrease in antibody levels during the first months after SARS-CoV-2 infection and even in the early convalescent phase^23,24^. A recent study suggested as independent factors associated with stability of antibodies over time the high-risk exposure to COVID-19 patients and a history of symptomatic COVID-19^24^. We did not find a statistically significant association neither with professional categories nor with previous history or duration of symptoms, although antibody levels measured during disease were associated with more severe symptoms, probably with also a causal effect^25^. However, lack of significance was conditioned by the small subgroup sample size, and, interestingly, none of the subjects with a RT-PCR positivity period longer than 3 weeks resulted in seronegative.

We searched for characteristics associated with previous SARS-CoV-2 positivity and, in subjects negative to a previous RT-PCR, we found an excess of subjects vaccinated for influenza or BCG or with a previous TB. This finding is in line with current literature, suggesting a role for non-COVID-19 vaccinations in decreasing SARS-CoV-2 infection risk, probably by enhancing non-specific innate response (trained immunity)^26–28^. However further studies are needed to confirm this association. We also found an association between positivity and chronic disease or high BMI levels, which appears to be related to increased infection risk in subjects with these chronic conditions. We also found an association with high CRP levels. Although BMI and CRP levels are generally linked, we found independent associations in our population. Moreover, a recent Mendelian randomization study showed a genetic correlation between COVID-19 and BMI but not CRP levels^29^. Furthermore, we finally found an association between previous infection and low neutrophils levels. High CRP and low neutrophil levels were found in COVID-19 subjects during the acute phase, mainly in severe cases^30,31^, and even in the first months after recovery, due to the persistency of an unresolved inflammation^20^. This unresolved inflammatory status could be suggested as one of the mechanisms underlying post-acute sequelae of COVID-19 (PASC) and it could be present in recovered subjects also in absence of symptoms^20^.

Interestingly, CRP is associated with baseline IgG levels, suggesting that the protection potential from further SARS-CoV-2 exposure is proportional to this persistent baseline inflammation status. This study suggests considering post-infection, rather than post-Covid19 baseline inflammatory status as an underlying pathway that could mark the extent of the specific immune response. Since its potential involvement in the etiopathogenesis of PASC, this result could be useful for the search of the related underlying unresolved conditions. The findings related to red blood cell markers, suggested as potentially involved in the SARS-CoV-2-dependent effect^32^, need to be further studied, however, they could strengthen this hypothesis. MCHC levels were reported to decrease the following onset of disease^33^, probably due to iron reduction following inflammation^34^.

We finally investigated if the best performing pattern could be defined in the population. We identified three patterns of IgG kinetics in the early immunization phases, characterized by different pre-vaccine IgG levels (negative, low, and high), showing different IgG increasing rate mainly up to T1, then conditioning the rapid achievement of ideal IgG levels in the early period. We did not find a specific behavior for positive subjects. In fact, our results showed similar kinetics for subjects with undetectable IgG at baseline, independent of previous exposure to SARS-CoV-2. Moreover, rapid growth after the first dose was observed in those with past exposure to SARS-CoV-2 and high pre-vaccine antibody levels (higher than 50 AU/ml with the used test). Although it is well known that one SARS-CoV-2 mRNA vaccine dose is sufficient to increase both cellular and humoral immune response in COVID-19–recovered subjects, this study suggests considering baseline IgG levels as a predictive marker of the extent of the early SARS-CoV-2 vaccine response in SARS-CoV-2 positive subjects. Serologic testing for defining the presence of anti-SARS-CoV-2 antibodies titer is not recommended before vaccination. However, our data suggest that measuring baseline IgG values could be useful in subgroups of positive subjects with the indication for a single dose when they are at higher risk of being infected by variants of concern or with expected lower vaccine response^35^.

This study has some limitations. The observational design does not allow a cause-effect interpretation of the observed relationship between previous exposure to SARS-CoV-2 and biomarkers levels, as well as between biomarkers levels and vaccine response. Moreover, this study was based on vaccine antibody response, then only on antibodies produced by plasma cells, without testing for IgG neutralization potential. Although antibodies are a central component of vaccine efficacy, memory B and T cells may be important for long-term protection^36^. However, during the early immunization period, B and T cell responses relate to serological responses for SARS-CoV-2 vaccines^36^, therefore IgG kinetics could be considered representative of vaccine response. The main strength of this study is the availability of a set of clinical and laboratory biomarkers since most studies reported only IgG determinations without collecting further data. Moreover, the use of a pattern analysis allowed identifying the real patterns existent in the population, discriminating among subjects with previous exposure to SARS-CoV-2 those with a rapid vaccine response after the first dose, which could be useful for dose prioritization decisions.

This work suggested post-infection unresolved inflammatory status as factors associated with IgG anti-SARS-CoV-2, in subjects previously exposed to SARS-CoV-2. This finding could help to characterize the nature of unresolved inflammation post-SARS-Cov-2 infection, potentially involved in PASC^20^. Moreover, already available CRP levels could help to predict IgG levels. As for SARS-CoV-2 mRNA vaccination response, in our population, subjects with a previous natural SARS-CoV-2 infection did not show a consistent behavior, considering neither vaccine-independent antibody levels nor post-vaccination kinetic patterns.

## Methods

### Study population

The study population consisted of workers of the healthcare agency ASST Sette Laghi (Varese, Italy), who underwent voluntary vaccination with COVID-19 mRNA BNT162b2. From the whole population of healthcare workers (N = 5143), a random sample of 431 respondents was invited to monitor their serum levels of IgG anti*-*SARS-CoV-2 following vaccination. A total of 175 workers (40.6%; 14 physicians, 111 nurses, 27 nurse assistants, and 23 administrative clerks; 68 having worked in a COVID-19 unit) finally adhered. Among these, 137 participants (78.9%) received both the first and the second vaccine dose (21 days between doses) within 2 months from the start of the vaccination campaign and were included in the analysis of vaccine response kinetics. The study was approved by the Ethic Committee of the ASST Sette Laghi (69/2020, 2020 November 17th) and informed consent was signed by participants. All methods were performed in accordance with the relevant guidelines and regulations.

### Data collection

At T0, a web-based questionnaire was sent, addressing participants' general information, lifestyle, and occupational risk factors. According to Italian law, privacy and data safety will be implemented and guaranteed by up-to-date encryption standards. Available clinical records were also collected in the hospital upon informed consent signature. A subject was defined symptomatic where dyspnea or fever > *37.5* °C or loss of taste or smell were reported. Where other flu-like symptoms were reported, such as runny nose, headaches, or a dry cough, subjects were categorized as pauci-symptomatic. Finally, a blood sample was drawn for the assay of SARS-CoV-2 IgG levels at three-time points: pre-vaccine baseline (the day before the date of vaccination—T0), three weeks after the first dose (the day before the 2nd dose—T1), and 2 weeks after the 2nd dose (T2).

### Laboratory analyses

At T0, T1, and T2, a blood sample was collected. For the determination of IgG anti–SARS-CoV-2, an indirect chemiluminescence immunoassay method (LIAISON^®^ DiaSorin)^37^ was used to determine SARS-CoV-2 S1/S2 specific IgG. The test sensitivity is 90.4% (79.4–95.8%) and its specificity is 98.5% (97.5–99.2%), as reported by the manufacturer. The measuring range spanned from 3.8 to 400.0 AU/mL; values higher than 15.0 AU/mL were considered positive. In case of a value higher than the upper limit of the test (400 AU/ml), to estimate the real IgG levels, we repeated the test after dilutions on serum aliquots prepared and conserved in a biobank from blood samples collected at the same time. The biobank procedures met the international standards^38^ and the national guidelines and specific consent was obtained from all participants.

At T0, blood samples were also analyzed in the centralized Hospital Laboratory for blood cell count, serum lipids, creatinine, gamma-glutamyl transferase (GGT), transaminases, lactic acid dehydrogenase (LDH), ultra-sensitive C-reactive protein (CRP), and high-sensitive troponin T levels, using commercial reagents and automatic analyzers. Low-density lipoprotein (LDL)-cholesterol was calculated using the Friedewald formula.

### Statistical analyses

We first summarized demographic and clinical characteristics, as well as of markers distributions, at baseline (T0) according to four different groups: negative real-time reverse transcriptase-polymerase chain reaction (RT-PCR) and negative IgG (below 15 AU/ml); negative RT-PCR and positive IgG; positive RT-PCR and positive IgG; and positive RT-PCR with negative IgG. Summary statistics included mean and standard deviation for normally distributed continuous variables; median and interquartile range for skewed continuous variables; and absolute and relative frequencies for discrete variables. Homogeneity across groups was tested using t-test, Wilcoxon rank test, or chi-square test, respectively. The same analyses were repeated among the subgroup of subjects undergoing vaccination and with longitudinal data on serum IgG. Then, we explored the cross-sectional association between demographic characteristics and circulating biomarkers with serum IgG levels (log-transformed) at T0, with linear regression models (standardized coefficients, per 1 SD increase), in SARS-Cov-2 IgG-positive subjects and stratifying for clinical history.

Among subjects undergoing vaccination, we modeled the IgG level progression over time using a group-based approach for normally distributed data with the Proc Traj procedure in SAS software^39^. This method assumes that several subgroups exist in a population, which are characterized based on their longitudinal outcome patterns. Each individual is then assigned to one group, based on posterior probabilities. Due to the skewed distribution of serum IgG (especially after vaccination), the trajectory analyses were conducted on the log-transformed variable. All levels higher than the LOD were used. As recommended^40^, we first selected the number of groups (from 1 to 4) by fixing second-order equations for all groups; hence, we selected the shape of the pattern of change for each group over time. In both steps, selection criteria were based on the difference in Bayesian Information Criterion (BIC) between two models^40^. According to these, we selected a 3-groups model: group A and group B characterized by a linear pattern with a similar slope and different intercept at T0; and group C in which the IgG time progression followed a quadratic function. Finally, we characterized the three groups identified with the trajectory analyses with respect to their demographic, clinical, and biomarkers characteristics, at first through univariate analyses. For those characteristics showing an association, we used a generalized logistic model (Group A as reference) to estimate the prevalence odds ratios for belonging to group B or C, in a multivariate fashion. All the analyses were conducted using SAS 9.4 release.

## Supplementary Information

Supplementary Information.

## Data Availability

The datasets generated and analysed during the current study are available from the corresponding author on reasonable request.
