# Supplementary material for: Anti-SARS-CoV-2 antibody levels and kinetics of vaccine response: potential role for unresolved inflammation following recovery from SARS-CoV-2 infection
Source: Sci Rep. 2022 Jan 10;12:385. doi: 10.1038/s41598-021-04344-y (PMC8749002; doi:10.1038/s41598-021-04344-y)
Supplement: Supplementary file 1 — Supplementary Information. [file 41598_2021_4344_MOESM1_ESM.docx]

**Anti-SARS-CoV-2 antibody levels and kinetics of vaccine response. Potential role for unresolved inflammation following recovery from SARS-CoV-2 infection (**Gianfagna F. *et al*.)

**SUPPLEMENTARY MATERIALS**

**Table S1:** Association between IgG levels and clinical characteristics or circulating biomarkers among subjects with previous exposure to SARS-CoV-2 (positive to previous RT-PCR or to IgG at baseline; N=75).

|  | Increase of 1 SD of baseline IgG (dependent variable) per 1 SD of the independent variable | |
| --- | --- | --- |
| Independent variable | Est±SE | p-value |
| **Demographic and clinical history** |  |  |
| Age (years) | 0.23±0.13 | 0.063 |
| Women | 0.62±0.37 | 0.090 |
| Previous SARS-CoV-2 positivity (RT-PCR) | 0.03±0.29 | 0.927 |
| BMI (Kg/m^2^) | 0.1±0.13 | 0.472 |
| Anti-tetanus vaccination (last 10 years) | -0.27±0.37 | 0.473 |
| Anti-influenza vaccination (last year) | -0.12±0.29 | 0.665 |
| BCG vaccination or PPD positivity | 0.15±0.27 | 0.579 |
| Autoimmune diseases | 0.33±0.35 | 0.335 |
| Chronic diseases ^§^ | 0.84±0.4 | 0.036 |
| **Organ damage biomarkers** |  |  |
| Creatinine (mg/dl) | -0.15±0.13 | 0.243 |
| Aspartate transaminase - AST (U/l) | 0.14±0.13 | 0.260 |
| Alanine transaminase - ALT (U/l) | -0.01±0.13 | 0.964 |
| Lactate dehydrogenase - LDH (U/l) | 0.06±0.14 | 0.666 |
| Troponin T (ng/l) | 0.14±0.13 | 0.280 |
| **Inflammatory biomarkers** |  |  |
| CRP (mg/l) | 0.33±0.12 | 0.007 |
| Leucocytes (10^9^cells/l) | -0.06±0.13 | 0.620 |
| Lymphocytes (10^9^cells/l) | -0.06±0.13 | 0.622 |
| Monocytes (10^9^cells/l) | 0.03±0.13 | 0.829 |
| Neutrophils (10^9^cells/l) | -0.06±0.13 | 0.620 |
| Platelets (10^9^cells/l) | -0.13±0.13 | 0.316 |
| Mean Platelet Volume - MPV (fl) | 0.07±0.13 | 0.590 |
| **Red blood cells** |  |  |
| Red blood cells (10^12^cells/l) | -0.25±0.13 | 0.051 |
| Mean Corpuscular Volume – MCV (fl) | -0.13±0.13 | 0.335 |
| Hematocrit (%) | -0.31±0.13 | 0.014 |
| RDW (%) | 0.31±0.13 | 0.014 |
| Hemoglobin (g/dl) | -0.33±0.13 | 0.008 |
| MCHC (g/dl) | -0.25±0.13 | 0.047 |

**Figure S1:** Correlation between IgG levels and CRP values (Spearman r= 0.4, p-value =0.005) among subjects with (black triangle, N=75) or without (grey circle, N=100) previous exposure to SARS-CoV-2 (positivity to previous RT-PCR or to IgG at baseline).

**
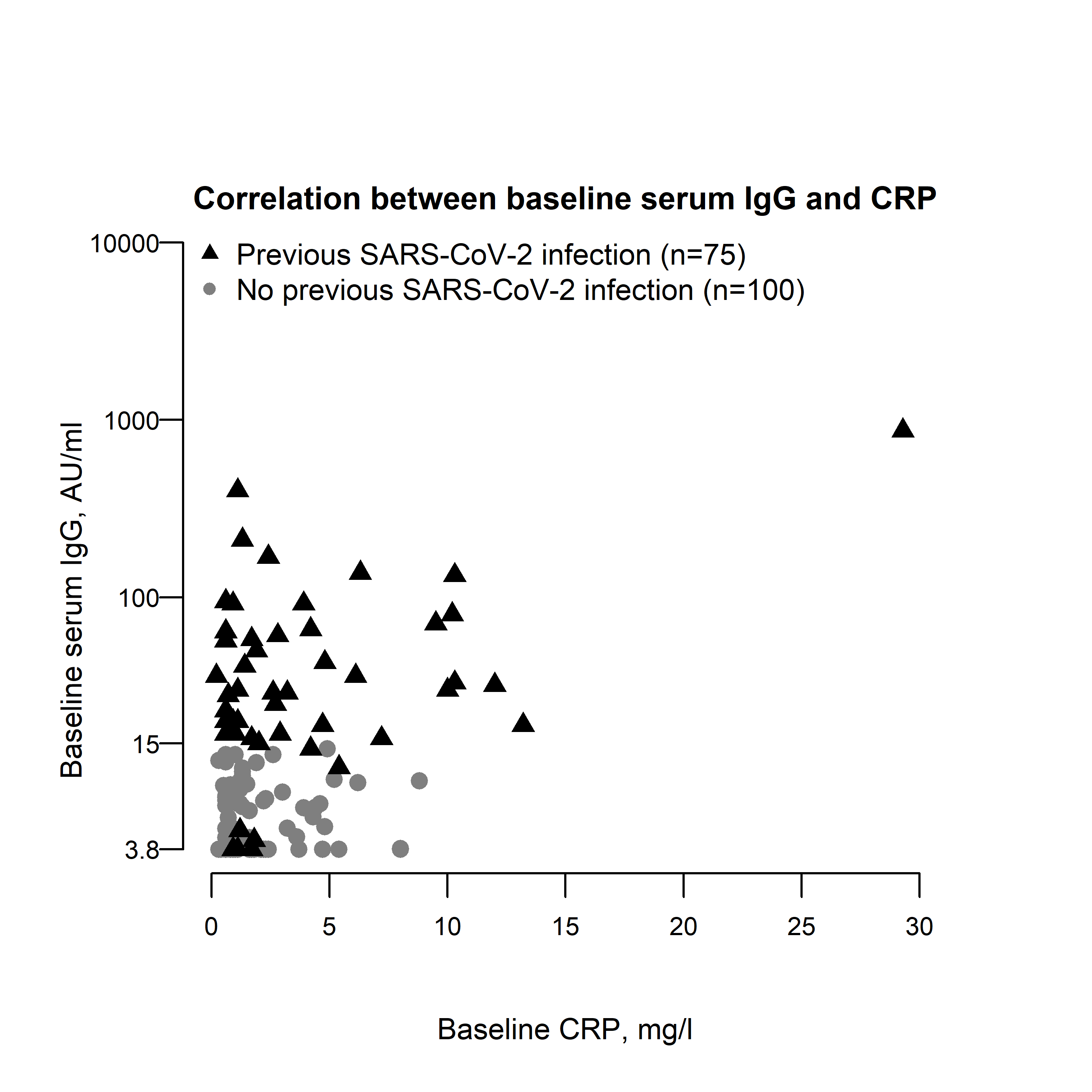
**

**Table S2:** Population characteristics by previous SARS-CoV-2 positivity and IgG levels at baseline, among the n=137 subjects undergoing vaccination/with longitudinal data. Mean (SD) for continuous variables, n (%) for categorical variables.

|  | **Negative RT-PCR** | | **Positive RT-PCR** | | **p-value^1^** | **p-value^2^** |
| --- | --- | --- | --- | --- | --- | --- |
|  | **A. IgG negative** | **B. IgG positive** | **C. IgG positive** | **D. IgG negative** |  |  |
| N | 86 | 22 | 21 | 8 | - | - |
| Baseline anti-S1/S2 IgG (AU/ml)* | 4.8 (3.8, 8) | 29 (17, 66) | 36 (28, 92) | 4 (3.8, 7.9) | 0.0003 | <.0001 |
| **Demographic and clinical history** |  |  |  |  |  |  |
| Age (years) | 47.6 (8.5) | 50.2 (8.1) | 44 (10.5) | 47.1 (12.5) | 0.09 | 0.78 |
| Women | 77 (89.5%) | 22 (100%) | 17 (81%) | 5 (62.5%) | 0.02 | 0.57 |
| Body mass index (Kg/m^2^)* | 23 (20.3,27) | 23.9 (19.9,28.2) | 24.8 (22.7,27.4) | 25.1 (23.8,26.9) | 0.06 | 0.05 |
| Anti-tetanus vacc. (last 10 years) | 15 (17.4%) | 2 (9.1%) | 2 (9.5%) | 2 (25%) | 0.80 | 0.37 |
| Anti-influenza vacc. (last year) | 27 (31.4%) | 12 (54.5%) | 4 (19%) | 3 (37.5%) | 0.23 | 0.48 |
| BCG vaccination or PPD positivity | 32 (37.2%) | 10 (45.5%) | 4 (19%) | 1 (12.5%) | 0.03 | 0.35 |
| Autoimmune diseases | 57 (66.3%) | 17 (77.3%) | 10 (47.6%) | 5 (62.5%) | 0.05 | 0.23 |
| Chronic diseases^§^ | 16 (18.6%) | 2 (9.1%) | 5 (23.8%) | 0 (0%) | 0.94 | 0.46 |
| **Organ damage biomarkers** |  |  |  |  |  |  |
| Creatinine (mg/dl) | 0.9 (0.1) | 0.8 (0.1) | 0.9 (0.1) | 0.9 (0.2) | 0.57 | 0.39 |
| Aspartate transam. - AST (U/l) | 21.4 (7) | 24.3 (8.8) | 24.7 (5.4) | 21 (7.6) | 0.28 | 0.05 |
| Alanine transam. - ALT (U/l) | 24.2 (15.1) | 28.9 (17.3) | 29.9 (15.9) | 27.1 (13.3) | 0.23 | 0.08 |
| Lactate dehydrogenase - LDH (U/l) | 190 (34.3) | 200.2 (29) | 206.1 (49.9) | 188.6 (21.1) | 0.27 | 0.11 |
| Troponin T (ng/l) | 4.4 (4.4) | 4.7 (2.9) | 4.5 (2.4) | 4.8 (2.2) | 0.93 | 0.74 |
| **Inflammatory biomarkers** |  |  |  |  |  |  |
| CRP (mg/l) | 1.9 (1.9) | 2.9 (2.9) | 5.8 (7) | 2.3 (1.8) | 0.0003 | 0.001 |
| Leucocytes (10^9^cells/l) | 7.1 (1.7) | 7.1 (2.4) | 5.8 (1.5) | 6.3 (1.3) | 0.003 | 0.05 |
| Lymphocytes (10^9^cells/l) | 2.1 (0.7) | 2.2 (0.8) | 1.8 (0.7) | 1.8 (0.5) | 0.03 | 0.17 |
| Monocytes (10^9^cells/l) | 0.5 (0.2) | 0.6 (0.2) | 0.5 (0.2) | 0.5 (0.2) | 0.62 | 0.67 |
| Neutrophils (10^9^cells/l) | 4.2 (1.3) | 4.2 (1.8) | 3.2 (1.2) | 3.9 (1.3) | 0.01 | 0.06 |
| Platelets (10^9^cells/l) | 267.5 (51.9) | 268.9 (54.2) | 258.1 (70.7) | 261.9 (58.5) | 0.46 | 0.67 |
| Mean Platelet Volume - MPV (fl) | 10.7 (0.8) | 10.8 (1) | 10.6 (0.8) | 10.5 (0.7) | 0.39 | 0.67 |
| **Red blood cells** |  |  |  |  |  |  |
| Red blood cells (10^12^cells/l) | 4.7 (0.5) | 4.6 (0.4) | 4.7 (0.6) | 4.9 (0.4) | 0.73 | 0.66 |
| Mean Corp. Vol. – MCV (fl) | 87.3 (5.4) | 87.7 (5.4) | 84.5 (8.9) | 88.8 (2.8) | 0.20 | 0.50 |
| Hematocrit (%) | 41.2 (3.5) | 40.5 (4) | 39.4 (5.5) | 43.7 (3.2) | 0.64 | 0.42 |
| Red Distribution Width – RDW (%) | 12.9 (1) | 13.2 (1.1) | 13.8 (2.5) | 12.4 (0.8) | 0.15 | 0.10 |
| Hemoglobin (g/dl) | 13.9 (1.3) | 13.5 (1.5) | 13.2 (2.4) | 14.9 (1.4) | 0.71 | 0.32 |
| Mean Corp. Hem. Conc.-MCHC(g/dl) | 33.7 (1) | 33.3 (1) | 33.4 (1.9) | 33.9 (0.8) | 0.57 | 0.12 |

Abbreviations: PPD, Purified Protein Derivative test (tuberculin test).
°: history of transplant, hematological diseases, multiple sclerosis, oncologic diseases, HIV.
§: diabetes mellitus, chronic renal failure, hypothyroidism.

In the Table: either mean (SD) or median (25-75°percentiles, as indicated with an *) for continuous variables; and n (%) for categorical variables.
P-value: comparison across study populations, as defined below. Continuous variables: t-test (in occurrence of mean and SD) or Wilcoxon rank test (median and IQR). Categorical variables: chi-square tests
1: comparison between negative (n=108) and positive (n=29) RT-PCR [columns A and B vs. C and D]
2: comparison between negative RT-PCR with negative Ab (n=86), and either positive Ab or positive RT-PCR (n=51) [column A vs. B, C and D]

**Table S3:** Characteristics of subjects belonging to the identified trajectories, according to their previous RT-PCR positivity to SARS-CoV-2, IgG levels at baseline, CRP levels (higher or lower than 5 mg/l) among the n=137 subjects undergoing vaccination/with longitudinal data.

| **Baseline characteristics** | | **Trajectory Group A (N=101)** | **Trajectory Group B (N=22)** | **Trajectory Group C (N=14)** | **Total** |
| --- | --- | --- | --- | --- | --- |
|  |  |  |  |  |  |
| No previous exposure | Negative RT-PCR, Negative IgG | 84 (97.7%) | 2 (2.3%) | 0 (0%) | 86 (100%) |
| Previous exposure to SARS-CoV-2 | Negative RT-PCR, Positive IgG | 10 (45.5%) | 6 (27.3%) | 6 (27.3%) | 22 (100%) |
|  | Positive RT-PCR, Positive IgG | 1 (4.8%) | 13 (61.9%) | 7 (33.3%) | 21 (100%) |
|  | Positive RT-PCR, Negative IgG | 6 (75%) | 1 (12.5%) | 1 (12.5%) | 8 (100%) |
|  |  |  |  |  |  |
| No previous exposure | Negative RT-PCR and IgG, CRP <=5 | 78 (97.5%) | 2 (2.5%) | 0 (0%) | 80 (100%) |
|  | Negative RT-PCR and IgG, CRP > 5 | 6 (100%) | 0 (0%) | 0 (0%) | 6 (100%) |
| Previous exposure to SARS-CoV-2 | Positive RT-PCR or IgG, CRP<=5 | 16 (41.0%) | 12 (30.8%) | 11 (28.2%) | 39 (100%) |
|  | Positive RT-PCR or IgG, CRP>5 | 1 (8.3%) | 8 (66.7%) | 3 (25%) | 12 (100%) |
|  |  |  |  |  |  |
